# Increased chromosomal radiosensitivity in asymptomatic carriers of a heterozygous *BRCA1* mutation

**DOI:** 10.1186/s13058-016-0709-1

**Published:** 2016-05-17

**Authors:** Annelot Baert, Julie Depuydt, Tom Van Maerken, Bruce Poppe, Fransiska Malfait, Katrien Storm, Jenneke van den Ende, Tim Van Damme, Sylvia De Nobele, Gianpaolo Perletti, Kim De Leeneer, Kathleen B. M. Claes, Anne Vral

**Affiliations:** Department of Basic Medical Sciences, Ghent University, Ghent, Belgium; Center for Medical Genetics, Ghent University Hospital, Ghent, Belgium; Department of Medical Genetics, University of Antwerp/University Hospital of Antwerp, Antwerp, Belgium; Biomedical Research Division, Department of Theoretical and Applied Sciences, University of Insubria, Busto Arsizio, Italy

**Keywords:** *BRCA1* mutations, DNA damage repair, Homologous recombination, G_2_/M cell-cycle checkpoint, Ionizing radiation, G_2_ micronucleus assay, Radiosensitivity indicator, Nonsense-mediated decay, Haploinsufficiency

## Abstract

**Background:**

Breast cancer risk increases drastically in individuals carrying a germline *BRCA1* mutation. The exposure to ionizing radiation for diagnostic or therapeutic purposes of *BRCA1* mutation carriers is counterintuitive, since BRCA1 is active in the DNA damage response pathway. The aim of this study was to investigate whether healthy *BRCA1* mutations carriers demonstrate an increased radiosensitivity compared with healthy individuals.

**Methods:**

We defined a novel radiosensitivity indicator (RIND) based on two endpoints measured by the G_2_ micronucleus assay, reflecting defects in DNA repair and G_2_ arrest capacity after exposure to doses of 2 or 4 Gy. We investigated if a correlation between the RIND score and nonsense-mediated decay (NMD) could be established.

**Results:**

We found significantly increased radiosensitivity in the cohort of healthy *BRCA1* mutation carriers compared with healthy controls. In addition, our analysis showed a significantly different distribution over the RIND scores (*p* = 0.034, Fisher’s exact test) for healthy *BRCA1* mutation carriers compared with non-carriers: 72 % of mutation carriers showed a radiosensitive phenotype (RIND score 1–4), whereas 72 % of the healthy volunteers showed no radiosensitivity (RIND score 0). Furthermore, 28 % of *BRCA1* mutation carriers had a RIND score of 3 or 4 (not observed in control subjects). The radiosensitive phenotype was similar for relatives within several families, but not for unrelated individuals carrying the same mutation. The median RIND score was higher in patients with a mutation leading to a premature termination codon (PTC) located in the central part of the gene than in patients with a germline mutation in the 5′ end of the gene.

**Conclusions:**

We show that *BRCA1* mutations are associated with a radiosensitive phenotype related to a compromised DNA repair and G_2_ arrest capacity after exposure to either 2 or 4 Gy. Our study confirms that haploinsufficiency is the mechanism involved in radiosensitivity in patients with a PTC allele, but it suggests that further research is needed to evaluate alternative mechanisms for mutations not subjected to NMD.

**Electronic supplementary material:**

The online version of this article (doi:10.1186/s13058-016-0709-1) contains supplementary material, which is available to authorized users.

## Background

Breast cancer (BC) is the most common malignancy in the Western world (http://www.who.int/cancer/detection/breastcancer/en/). Approximately 15 % of all patients with BC have at least one relative affected by BC. About 15 % of all familial BCs can be attributed to a mutation in the *BRCA1* or *BRCA2* gene [1]. Since the discovery of BRCA1, many different functions have been attributed to this protein. In its function as a tumour suppressor gene, BRCA1 plays a crucial role in DNA double-strand break (DSB) repair pathways (reviewed in [2, 3]). BRCA1 is, for instance, important in homologous recombination (HR), a pathway for repair of DSB in late S and G_2_ phases of the cell cycle [4–6]. BRCA1 also plays an important role in the G_2_/M checkpoint control, allowing the cell to repair DNA damage before proceeding to the next phase of the cell cycle [7].

Carriers of a heterozygous *BRCA1* mutation may show enhanced radiosensitivity associated with an increased carcinogenic risk after exposure to diagnostic or therapeutic ionizing radiation (IR). Several studies have shown that exposure to diagnostic X-rays may cause cancer in healthy *BRCA1* mutation carriers [8–11], whereas researchers in other studies could not detect a positive association between exposure to IR and breast cancer risk in *BRCA1/2* mutation carriers [12–15]. Also, researchers analysing the impact of (adjuvant) radiotherapy on breast cancer risk in *BRCA1* and *BRCA2* mutation carriers reported no univocal conclusion [16]. The contradictory data obtained in these studies are due mainly to the constraints in the design of the performed studies.

Since long-term studies of the effect of exposure to IR in mutation carriers are difficult to set up and are reputed to be unethical, it is clear that more empiric data are needed to determine in vitro the radiosensitivity of patients carrying a germline mutation. To date, research has yielded contradictory results [17–26].

The G_0_ micronucleus assay performed on peripheral blood lymphocytes exposed to in vitro doses of 2 to 4 Gy is frequently used to assess chromosomal radiosensitivity. However, this assay is not optimized to detect defects in DSB repair activated during the G_2_ phase of the cell cycle or the G_2_/M checkpoint, two processes in which BRCA1 plays a major role because irradiation takes place in the G_0_ phase of the cell cycle.

We previously reported a modified micronucleus (MN) assay optimized to detect defects in the S or G_2_ phase of the cell cycle. This assay efficiently detected increased radiosensitivity in a patient with a mild form of ataxia-telangiectasia (A-T) and in heterozygous relatives [27].

In the present study, we applied the G_2_ micronucleus assay to further elucidate whether healthy *BRCA1* mutation carriers are characterized by an increased in vitro radiosensitivity. The endpoints of the study were (1) micronucleus yields and (2) G_2_/M checkpoint efficiency ratio. Both endpoints were assessed after irradiating phytohaemagglutinin (PHA)-stimulated peripheral blood lymphocytes with doses of 2 Gy and 4 Gy. With this assay, we assessed the mean differences in radiosensitivity for heterozygous *BRCA1* mutation carriers compared with healthy volunteers. We also scored the overall radiosensitivity in each mutation carrier using a radiosensitivity indicator (RIND) scoring system. In addition, as our *BRCA1* population consisted of individuals carrying different *BRCA1* mutations, we investigated if there was a link between radiosensitivity and the degree of nonsense-mediated decay (NMD) of the specific mutant allele.

## Methods

### Sample collection

Blood samples were collected from individuals consulting the clinic of the Centre for Medical Genetics, Ghent University Hospital, Belgium (CMGG). Both ethylenediaminetetraacetic acid (EDTA) and heparin blood samples were collected. EDTA samples were used for mutation analysis at CMGG, whereas the G_2_ MN assay was performed on heparinized blood samples. In addition, we collected heparinized blood samples from healthy volunteers (*n* = 20) without a personal or family history of BC to determine the normal distribution of micronucleus yields in controls. Lymphocytes were isolated from the blood samples using Lymphoprep™ (STEMCELL Technologies, Vancouver, BC, Canada) and were preserved in liquid nitrogen for analysis of NMD of the mutant allele. For a number of mutation carriers (*n* = 4) and healthy volunteers (*n* = 7), a second blood sample was taken to determine the reproducibility of the results obtained with the G_2_ MN assay at different time points.

This study was approved by the ethics committee of the Ghent University Hospital (B670201111641 d.d. 20/09/2011). All study participants (*n* = 18) were counselled by clinical geneticists in the context of a predictive (*n* = 16) or diagnostic (*n* = 2) test for hereditary BC and signed an informed consent form. Two of the eighteen *BRCA1* mutation carriers had developed BC, but their cancer treatment had finished more than 2 years ago; these women are both carriers of a substitution affecting the start codon (M01 and M02). The mean ages of the mutation carriers and healthy volunteers were 40.9 and 35.4 years, respectively (*p* = 0.26, *t* test).

### Molecular analysis

All patients selected for this study had a family history of breast and/or ovarian cancer and a *BRCA1* germline mutation. Targeted analysis for the familial mutation was performed by direct sequencing on two independently extracted DNA samples. No molecular analyses were performed in healthy volunteers, owing to absence of personal or familial anamnesis for BC.

### G_2_ micronucleus assay

For the G_2_ MN assay, a large blood culture was set up in the presence of the mitogen PHA. PHA stimulates T-lymphocyte division, resulting in a population of cycling lymphocytes (G_1_, S_1_, G_2_ and M phase) after 3 days of incubation, when the blood culture is irradiated. On the contrary, in the G_0_ MN assay, blood is first irradiated and then cultured in the presence of PHA, resulting in the irradiation of T lymphocytes in G_0_ phase. The addition of cytochalasin B (cyto B), an agent that blocks cytokinesis, to the blood cultures allows the identification of first-division cells as binucleated (BN) cells. A non- or misrepaired DSB can result in an acentric chromosomal fragment, which is detected as a micronucleus in the cytoplasm of the BN cell [17, 28].

**Fig. 1:**
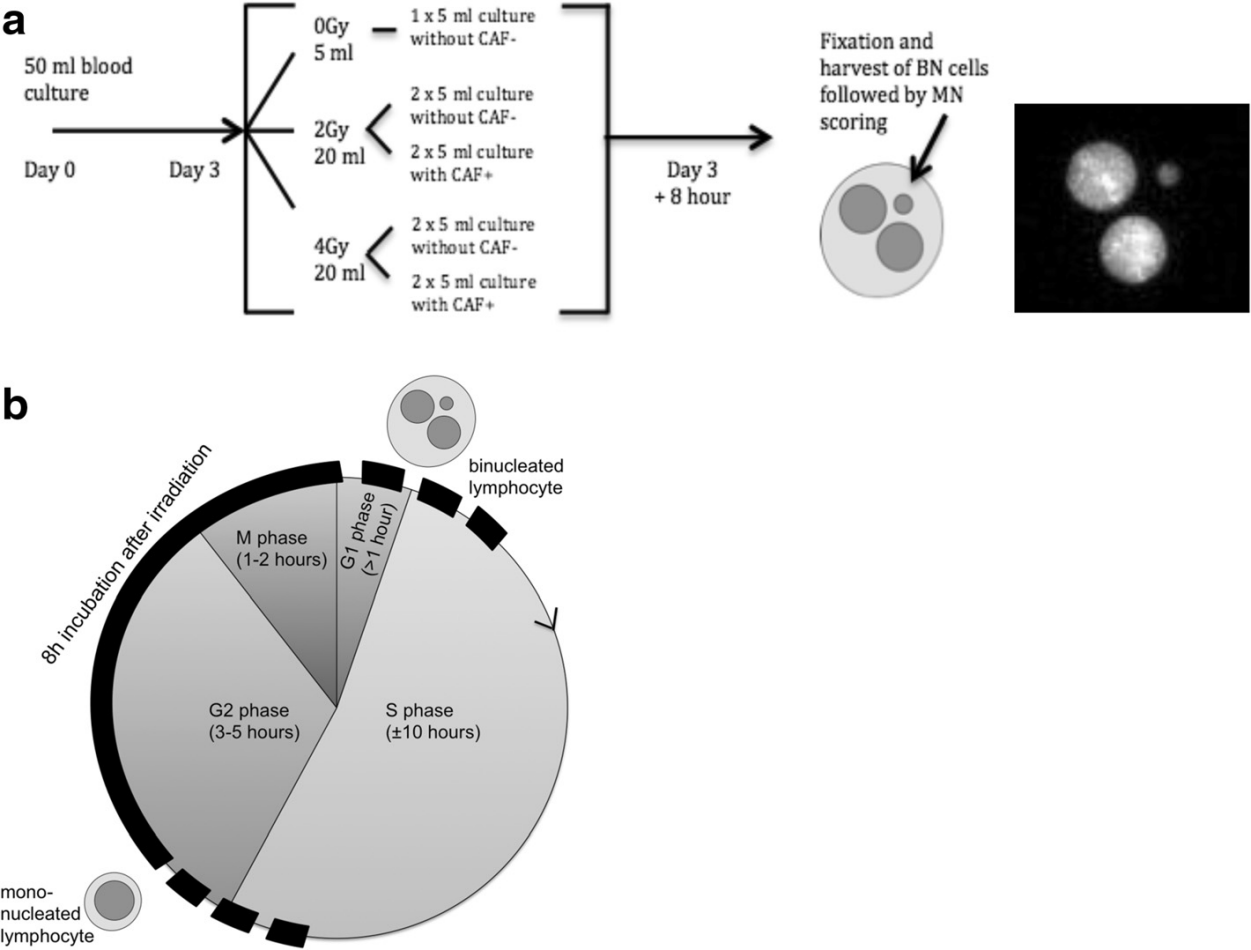
Design of the G_2_ micronucleus (MN) assay and overview of the cell cycle. **a** *Left*: Day 0: setup of one 50-ml large blood culture for every patient. Day 3: division of the cultures and subsequent irradiation. Addition of cytochalasin B and caffeine (CAF; if needed) immediately after irradiation, followed by incubation during 8 h before harvesting. *Right*: 4′,6-diamidino-2-phenylindole-stained binucleated cell with micronucleus generated with the Metafer4 system (MetaSystems). **b** Overview of the cell cycle for proliferating lymphocytes and approximate duration of the different phases with indication of the applied post-irradiation incubation period (*black*). At the start of irradiation, all lymphocytes in the blood culture were mononucleated. After a post-irradiation incubation of 8 h, the cultures were fixed and binucleated (BN) cells (cells that went through one mitosis) were scored for the presence of MN

More precisely, for the G_2_ MN assay, a 50-ml blood culture was set up using 5 ml of heparinized blood, 45 ml of complete RPMI (RPMI with 1 % l-glutamine and 0.5 % penicillin/streptomycin) containing 10 % foetal calf serum (FCS) and 1 ml of PHA (all Gibco®; Thermo Fisher, Rockford, IL) in a T75 culture flask [27]. This large culture was set up to avoid interculture variation during the first days of incubation. After 3 days, the culture was split, and sham irradiations as well as irradiations with 2 and 4 Gy ^60^Co gamma rays were performed. An overview of the experiments is shown in Fig. 1a. Immediately after irradiation, cyto B (6 μg/ml; Sigma-Aldrich, St. Louis, MO, USA) was added. To half of the irradiated cultures, caffeine (CAF, 4 mM; Sigma-Aldrich), an agent that abrogates the G_2_/M checkpoint, was added to determine the G_2_/M checkpoint efficiency ratio [29, 30]. On the basis of 5-bromo-2′-deoxyuridine (BrdU) results obtained in our previous study on ATM [27] and new experiments performed for this study, a post-irradiation incubation period of 8 h was selected for detecting DNA damage induced in cells in G_2_ phase (Fig. 1b). The cultures were then treated with 75 mM KCl and fixed once using a combination of methanol, acetic acid and Ringer’s solution (9 g NaCl + 0.42 g KCl + 0.24 g CaCl_2_ for 1 L of dH_2_O) (ratio 4/1/5, 4 °C) and thrice with methanol and acetic acid (ratio 4/1, 4 °C). Finally, the cell suspension was concentrated and spread on slides.

After 4′,6-diamidino-2-phenylindole staining, the slides were scanned with a Metafer 4 (MN score software module; MetaSystems; Altlussheim, Germany). This automated image analysis system selects BN cells and determines the number of MN per BN cell (see Fig. 1a). For each condition, two cultures were set up and two slides per culture were analysed. A minimum of 600 BN cells were scored on each coded slide for the presence of MN. BN and MN selected in the automated setting were manually checked for false-positives and false-negatives. To determine the G_2_/M checkpoint efficiency ratio, the quotient was determined for the MN yield obtained in the presence versus the absence of caffeine (MNCaf^+^/MNCaf^−^). The lower this ratio was, the more radiosensitive an individual was.

To assess the overall radiosensitive phenotype of each patient, a RIND score was calculated. The mean and standard deviation (SD) of the micronucleus yield and the G_2_/M checkpoint efficiency ratio assessed in the cohort of healthy volunteers served to determine cut-offs to define the radiosensitivity of individual *BRCA1* mutation carriers. An individual value equal to or greater than the mean MN yields in controls + 1 SD scored 1 point (colour-coded orange). A value equal to or greater than the mean MN yields of controls + 2 SD scored 2 points (colour-coded red). An individual value less than the mean of controls + 1 SD was scored as naught (colour-coded white). For the G_2_/M checkpoint, a similar conversion of the data was performed, with a value less than the mean checkpoint ratio assessed in controls − 1 SD scored 1 point (colour-coded orange) and the mean − 2 SD scored 2 points (color-coded red). An individual value greater than the mean of controls − 1 SD was scored as naught (colour-coded white). The combined scores from MN yields (0, 1 or 2 points) and checkpoint ratios (0, 1 or 2 points) were then added to form a single RIND score (range 0–4).

### Analysis of the cell-cycle phase at time of irradiation

BrdU was added to blood cultures of three individuals to analyse the phase of the cell cycle at the time of irradiation. BrdU (0.01 mM; Sigma-Aldrich) was added to the cultures immediately after irradiation. Binucleated cells that incorporated BrdU during synthesis were in S or G_1_ phase of the cell cycle at the moment of irradiation, whereas BrdU-negative binucleated cells were irradiated in G_2_ phase. We corrected for daughter nuclei incorporating BrdU after binucleation by adding BrdU 2 h before fixation to another subset of cultures. BrdU was visualized by fluorescence immunostaining with a monoclonal BrdU-specific antibody (M0744; Dako, Carpinteria, CA, USA).

### Analysis of the stability of the mutant allele

For this experiment, 2 × 10^6^ frozen lymphocytes were thawed and cultured in a mix of 1 ml of cRPMI, FCS (10 %), 2-mercaptoethanol (0.1 %, Gibco®) and sodium pyruvate (1 %; Life Technologies; Carlsbad, CA, USA). PHA (10 μl/ml) was added to stimulate cell division. At day 7, whole RNA was extracted using the QIAamp® RNeasy Mini Kit (QIAGEN, Valencia, CA, USA) according to the manufacturer’s instructions. Four hours before extraction, all cultures were split in two, and puromycin (200 μg/ml; Sigma-Aldrich) was added to one part. Puromycin was added to avoid NMD, a pathway responsible for the degradation of aberrant mRNA [31].

The total RNA and purity was measured using the DropSense96 reader (TRINEAN, Gentbrugge, Belgium). The RNA was converted into complementary DNA (cDNA) using the iScript™ cDNA Synthesis Kit (Bio-Rad Laboratories, Hercules, CA, USA). When RNA was extracted from cultures with puromycin, the converted cDNA is referred to as *cDNAp*. Before this cDNA synthesis, samples were treated with DNase (Heat&Run Kit; ArcticZymes, Tromsø, Norway) to remove any possible remaining genomic DNA (gDNA).

To determine the ratio of the mutant *BRCA1* allele versus the wild-type (WT) allele at the mRNA level, we performed polymerase chain reaction (PCR) amplification and Sanger sequencing of the amplicon harbouring the germline mutation and a heterozygous single-nucleotide polymorphism (SNP) (c.2311 T > C), if present. An overview of the primers used can be found in Additional file 1. Sanger sequencing was performed using the BigDye® Terminator Cycle Sequencing Kit (Life Technologies). PCR was followed by ethanol precipitation, and fragments were dissolved in a mix of Hi-Di™ formamide (10 μl; Life Technologies) and GeneScan™ 500 LIZ™ Size Standard (0.5 μl; Applied Biosystems, Foster City, CA, USA). The fragments were analysed on the ABI PRISM® 3730KL Genetic Analyzer (Life Technologies). Results were evaluated using GeneMapper® software (Applied Biosystems) for visual inspection and determination of the ratio of the peak heights representing the WT and mutant alleles, respectively (Additional files 2 and 3). Data were normalized with a control peak in the near vicinity. To confirm the results, the PCR amplicons were also sequenced with the sequencing by synthesis technology on a MiSeq instrument (Illumina, San Diego, CA, USA). The library preparation was performed using an adapted Nextera XT protocol (Illumina) as described by De Leeneer et al. [32]. Reads were mapped with CLC Genomics Workbench (CLC bio/QIAGEN, Aarhus, Denmark). The variant allele frequency (VAF) (which reflects the ratio of WT versus mutant allele) of both the mutation and a SNP (if available) was determined in the mutation carriers and in controls without a germline *BRCA1* mutation.

## Results

### Analysis of the cell-cycle phase at time of irradiation

On the basis of data reported in the literature, the cell cycle of proliferating lymphocytes takes between 18 and 22 h. The cell cycle is presented in Fig. 1b, and the mean length for each phase is indicated [33, 34]. With the BrdU immunostaining experiments, we determined that a post-irradiation incubation time of 8 h after 2- or 4-Gy irradiation resulted in blood cultures in which 80 % (±13 %) and 87 % (±3 %) binucleated cells, respectively, were in G_2_ phase (negative at BrdU staining) at the time of irradiation. In sham-irradiated samples, the percentage of BrdU-negative binucleated cells was much lower (56 ± 8 %). These observations suggest that synchronization of the cells in G_2_ phase took place because of radiation-induced G_2_ arrest. When caffeine, an agent that abrogates the G_2_/M checkpoint [29], was added to the cultures, the percentage of cells in G_2_ phase decreased (2 Gy 73 % ± 6 %; 4 Gy 79 % ± 2 %). The high percentage of cells irradiated in G_2_ phase indicates that the harvest of BN cells 8 h post-irradiation is optimal.

### Results of G_2_ MN assay

**Fig. 2:**
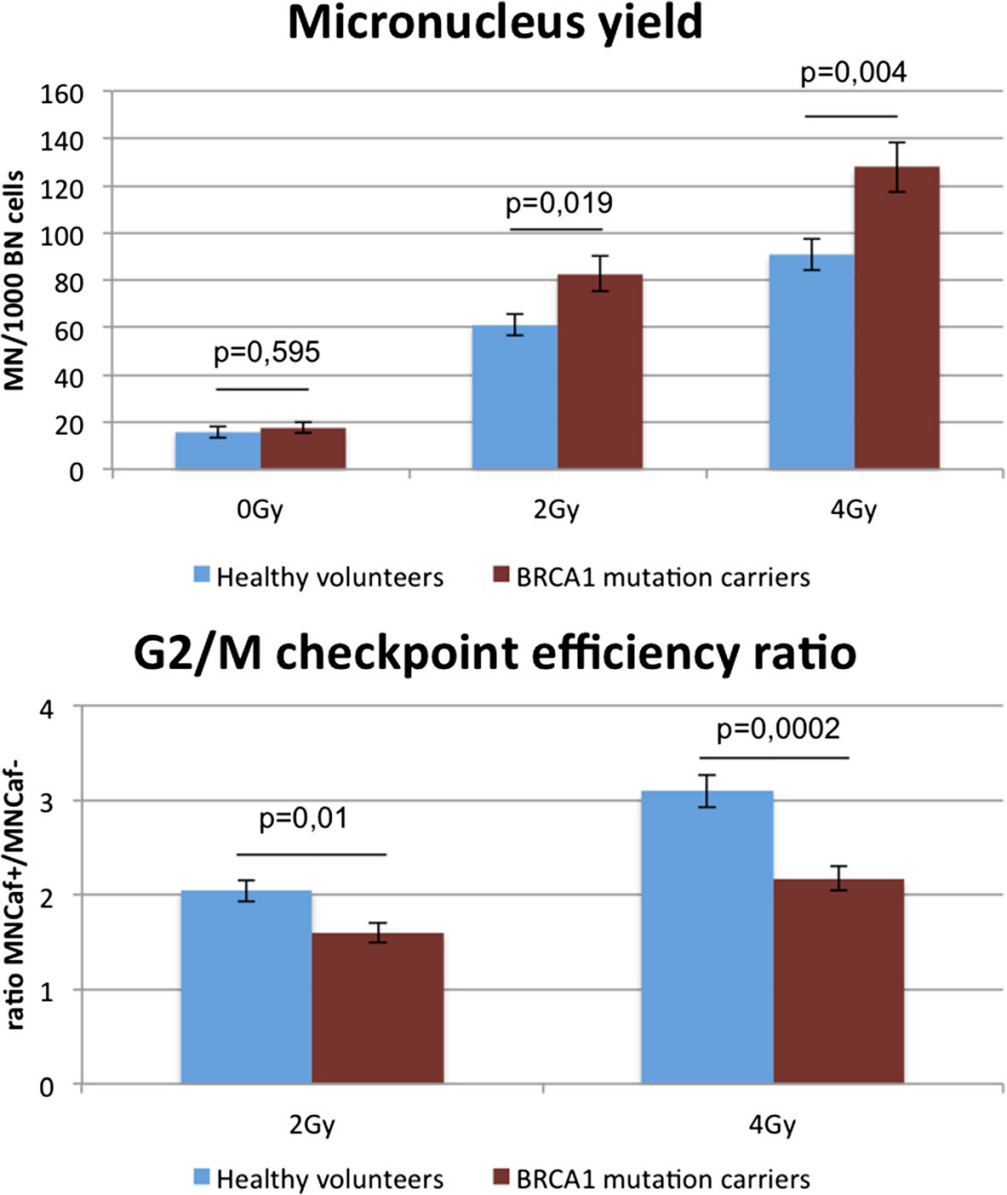
Mean micronucleus (MN) yields and G_2_/M checkpoint efficiency ratios obtained with the G_2_ MN assay in healthy volunteers and *BRCA1* mutation carriers. Significance was determined with a two-sided *t* test. *p* Values for each of the endpoints and dose points are indicated in the graph. Error bars represent the standard error of the mean. *BN* binucleated cell, *Caf* caffeine

The MN yield obtained for the sham-irradiated samples did not show a significantly different mean result between mutation carriers and healthy volunteers (Table 1 and Fig. 2). The mean values obtained for the radiosensitivity analysis are shown in Table 1 and Fig. 2. In *BRCA1* mutation carriers compared with healthy volunteers, a significantly increased radiosensitivity was observed for both endpoints at both radiation doses of 2 Gy and 4 Gy. Our results also show that the 4 Gy dose point was the most discriminative (highest significance based on *p* value) for both endpoints. We thus selected this dose to determine the individual overall radiosensitive phenotype. Results of the mutation analysis and individual results obtained for the two radiosensitivity endpoints upon 4 Gy irradiation are shown in Table 2. Each value received a colour code corresponding to a radiosensitivity score, from which a RIND score was calculated.

**Table 1:** Mean micronuclei yields and G_2_/M checkpoint efficiency ratios obtained with the G_2_ micronucleus assay

	Micronucleus yield (MN/1000 BN)			G_2_/M checkpoint efficiency (MNCAF^+^/MNCAF^−^)	
	0 Gy	2 Gy	4 Gy	2 Gy	4 Gy
Healthy volunteers					
Mean	15	61	91	2	3.1
SD	10	21	29	0.5	0.8
SEM	2	5	7	0.1	0.2
*BRCA1* mutation carriers					
Mean	16	83	128	1.6	2.2
SD	7	31	45	0.5	0.5
SEM	2	7	10	0.1	0.1
*p* Value (two-sided *t* test)	0.83	0.019	0.004	0.01	0.0002

*BN* binucleated cell, *CAF* caffeine, *MN* micronucleus or micronuclei, *SD* standard deviation

**Table 2:**
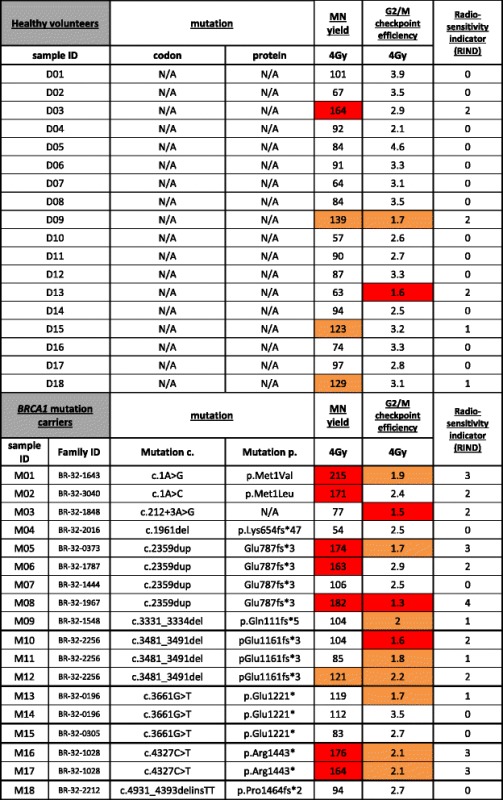
Results of mutation screening and radiosensitivity assessment for each individual

**Fig. 3:**
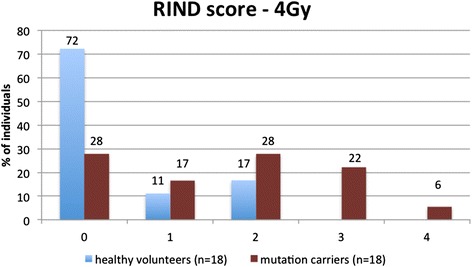
The distribution (%) of healthy volunteers and *BRCA1* mutation carriers over the different radiosensitivity indicator (RIND) scores

Significantly different RIND values were found for *BRCA1* mutation carriers (median 2) and healthy volunteers (median 0) (*p* = 0.0076, Mann-Whitney *U* test). Figure 3 shows the distribution of the *BRCA1* mutation carriers and healthy control subjects for the five different RIND scores. The distribution amongst the RIND scores between the two groups is significantly different (*p* = 0.034, Fisher’s exact test). The significantly different median and the significantly different distribution over the RIND scores obtained for both groups point towards a difference in response to radiation.

Repeated assessments were performed on blood specimens taken on two different occasions from a random sample of seven healthy volunteers and four mutation carriers to exclude intraindividual variations. Although minor variations were observed, no significant differences were observed between repeated measurements (*p* > 0.05 for both MN yield and G_2_/M checkpoint efficiency; repeated-measures analysis of variance).

We also investigated whether relatives of the subjects enrolled in this study, carrying the same mutation, had similar RIND scores. Individuals with the same family ID are known to be related (Table 2). For families BR-32-0196, BR-32-1028 and BR-32-2256, we had access to data and samples of several relatives. Both father and daughter of family BR-32-1028 showed a RIND score of 3. The siblings from family BR-32-2256 show RIND scores of 1 and 2, and we obtained scores of 0 and 1 for the third-degree relatives in family BR-32-0196. We thus conclude that there were no major differences in RIND scores within families. However, different RIND scores (ranging from 0–4) were observed between four individuals carrying the same mutation (c.2359dup), but we were unaware of a close relationship.

### Analysis of the stability of the mutant allele and correlation with the RIND score

The results of fragment analysis and massive parallel sequencing were comparable (Table 3). All results for cDNA, but not for gDNA, analyses are obtained by in duplicate experiments. The results of the MiSeq analysis are expressed as the VAF in percent; 50 % means equal expression of both alleles. The results of the fragment analysis are shown as a ratio of peak heights of mutant to WT allele. A value of 1 equals no loss of expression of the mutant allele. cDNA samples not treated with puromycin were scored as “evidence of NMD” (Table 3) when an average VAF ≤31 % or a ratio of peak heights <0.7 was obtained. We observed more variation for deletions/insertions than for substitutions. This can be explained by the more complex mapping of reads containing deletions/duplications. A large deletion and software struggling to map correctly to the reference sequence explains a VAF <50 % for gDNA in some patients (Table 3). Nevertheless, a drop in VAF for cDNA and not for cDNA with puromycin can still be distinguished.

**Table 3:** Stability of the mutant allele

		Fragment analysis	MiSeq (VAF)			
Donor	RIND score	Ratio peak height (mutant/WT allele)	cDNA	cDNAp	gDNA	Evidence for NMD?
M01 c.1A > G	3	0.7 (0.6–0.8)	49 (42–55)	52 (51–52)	49 (49–49)	No
M02 c.1A > C	2	/	/	/	/	No
M03 c.212 + 3A > G	2	/	/	/	/	No
M04 c.1961del	0	0.4 (0.4–0.4)	29 (25–33)	47 (43–51)	47 (47–47)	Yes
M05 c.2359dup	3	0.6 (0.6–0.6)	28 (25–31)	42 (38–45)	48 (48–48)	Yes
M06 c.2359dup	2	0.5 (0.5–0.5)	24 (23–25)	44 (41–47)	50 (50–50)	Yes
M07 c.2359dup	0	0.4 (0.4–0.5)	29 (27–30)	48 (30–67)	57 (55–59)	Yes
M08 c.2359dup	4	/	/	/	/	Yes
M09 c.3331_3334del	1	0.4 (0.4–0.4)	25 (18–31)	44 (34–48)	49 (49–49)	Yes
M10 c.3481_3491del	2	0.4 (0.4–0.4)	23 (16–30)	42 (28–52)	39 (32–46)^a^	Yes
M11 c.3481_3491del	1	0.4 (0.3–0.5)	23 (16–32)	41 (23–54)	39 (34–44)^a^	Yes
M12 c.3481_3491del	2	0.4 (0.4–0.4)	26 (16–34)	39 (28–50)	42 (35–49)^a^	Yes
M13 c.3661G > T	1	0.5 (0.3–0.6)	29 (28–30)	50 (41–57)	50 (46–53)	Yes
M14 c.3661G > T	0	0.4 (0.3–0.4)	31 (29–32)	52 (52–52)	50 (50–50)	Yes
M15 c.3661G > T	0	/	/	/	/	Yes
M16 c.4327C > T	3	0.5 (0.5–0.6)	24 (21–27)	51 (45–56)	/	Yes
M17 c.4327C > T	3	0.6 (0.4–0.7)	26 (21–30)	48 (44–53)	50 (50–50)	Yes
M18 c.4931_4393delinsTT	0	0.4 (0.3–0.4)	30 (28–31)	37 (28–47)	52 (52–52)	Yes

*cDNA* complementary DNA, *cDNAp* cDNA extracted in the presence of puromycin, *gDNA* genomic DNA, *NMD* nonsense mediated decay, *RIND* radiosensitivity indicator, *VAF* variant allele frequency, *WT* wild type

^a^VAF <50 % for gDNA

For M02, M08 and M15, no lymphocytes were available to perform this assay. However, as we had access to cDNA from other individuals with the same mutation, we were able to gain insight into the stability of the mutant mRNA for these three mutation carriers. For M16, no gDNA data could be obtained; information with regard to mutant mRNA stability for this mutation carrier was obtained via cDNAp and M17.

### Premature termination codon alleles in the central part of the gene

For all truncating mutations studied in the central part of the gene (7 unique mutations in 15 individuals), we found evidence for NMD. In general, 25–30 % residual truncated mRNA was detected in carriers of a premature termination codon (PTC) mutation. Ten of fifteen patients with a truncating allele showed a radiosensitive phenotype (RIND score 1–4, median 1).

### Mutations located in the 5′ part of the gene

For mutations in the 5′ part of the gene (*n* = 3), we have no evidence for NMD. The effect at the mRNA level for the c.212 + 3A > G splice-site mutation has previously been studied with quantitative PCR by our group. We showed that no full-length transcript is formed, but that it leads to a significantly increased expression of an alternative transcript (out of frame skip of 22 last nucleotides of exon 5; r.190_212del), which is not subjected to NMD [35, 36]. As M03 is not heterozygous for any of the tested common SNPs, results could not be re-analysed with the approach described in this paper. Our results for M03 showed a RIND score of 2, which can be attributed to a decreased G_2_/M checkpoint efficiency. For M01, carrier of the c.1A > G start codon mutation, no decline of the mutant allele could be detected when analysing the SNP data (Table 3). This suggests the conservation of the start codon or an alternative one. The mutation itself could not be quantified, owing to the presence of GC-rich areas near the start codon. The introduction of an alternative start codon could give rise to an aberrant protein with a dominant-negative effect [37]. Interestingly, both individuals carrying a mutation affecting the start codon had, respectively, RIND scores of 3 and 2. A median RIND score of 2 was observed for carriers of a mutation in the 5′ part of the gene.

## Discussion

Our study shows a significantly increased radiosensitivity in the group of healthy *BRCA1* mutation carriers compared with healthy controls for the two endpoints measured by the G_2_ MN assay for both doses of 2 and 4 Gy. Our results indicate that radiosensitivity in heterozygous *BRCA1* mutation carriers is a complex phenotype linked to defects in DNA damage repair, as well as to defective G_2_ arrest capacity. These results are in agreement with the studies performed by Pantelias and Terzoudi [30]. They applied the G_2_ chromatid break assay and reported that the G_2_/M checkpoint efficiency ratio is a good parameter for prediction of intrinsic radiosensitivity in A-T and cancer patients.

In vitro radiosensitivity has previously been investigated in patients with BC and *BRCA1* mutation carriers. Cardinale et al. recently published a meta-analysis combining all in vitro case-control studies in which the G_0_ MN assay on peripheral blood lymphocytes was used to analyse in vitro radiosensitivity in women with BC or with a known or putative genetic predisposition to BC [17]. Other cytogenetic assays, such as the chromosome aberration and G_2_ chromatid break assay or survival assays, have also been applied to determine radiosensitivity in a variety of cell types heterozygous for *BRCA1* mutations, using different irradiation protocols [18–26]. It is difficult, however, to correctly compare the results of these studies, as also concluded by Cardinale et al. [17], owing to different experimental set-ups to analyse in vitro radiosensitivity. However, despite the heterogeneity of the studies, most of the data generated, including ours, are suggestive of a different radiosensitive phenotype between *BRCA1* heterozygous mutant cells and control cells. Recent studies in which researchers investigated more specifically the functionality of the HR pathway in *BRCA1* heterozygous cells by means of γ-H2AX and RAD51 foci assays point towards a less efficient DSB repair by HR. These findings further support the evidence of increased radiosensitivity observed in *BRCA1* heterozygous cells when irradiated in the S or G_2_ phase of the cell cycle and are compatible with haploinsufficiency as the underlying mechanism [24, 25, 38]. The strength of our study is that radiosensitivity was analysed by means of two different endpoints obtained with a G_2_-specific MN assay developed by our group.

The radiosensitivity results in this study were obtained with doses of 2 and 4 Gy, which is considerably higher than any lifetime cumulative dose received by mammography screening. The average dose delivered to the breast glandular tissue per mammographic screening session is approximately 4 mGy [39]. Thus, direct extrapolation of our radiosensitivity results to the risks of mammography is not possible. Despite this limitation, our results may suggest caution in the IR exposure of healthy tissues of *BRCA1* mutation carriers for diagnostic purposes. It is furthermore noteworthy that recent studies have shown that 30-kV X-rays have a higher relative biological effect and are thus more harmful than conventional high-kilovoltage X-rays or ^60^Co gamma rays, on which current risk assessment is based. This implies that each mammogram may induce more DNA damage than commonly estimated [40, 41]. The authors of several papers have suggested that mammography screening might preferably be replaced by magnetic resonance imaging to avoid IR before the age of 30 years [16] or 40 years [42] in *BRCA1* mutation carriers. In addition, and more appropriately, given the high doses used in this assay, our results suggest caution in the use of adjuvant radiotherapy following breast-conserving surgery. In this respect, there is a need for well-designed studies to assess the incidence of second ipsi- or contralateral cancers upon adjuvant radiotherapy in mutation carriers [16].

Since it was our aim to develop a radiosensitivity assay applicable in a clinical setting, we performed the G_2_ MN assay on peripheral blood samples, which can easily be obtained during a genetic consult. Several studies have demonstrated that radiosensitivity of an individual is also detectable in cells of a type different from cells in which the tumour develops [18, 43, 44]. The scoring system with RIND scores varying between 0 and 4 allowed us to assess overall radiosensitivity due to both DNA repair and G_2_ arrest capacity of each mutation carrier (Table 2 and Fig. 3). With the help of this scoring system, we determined that 72 % of our healthy volunteers showed no radiosensitive phenotype. *BRCA1* mutation carriers, on the contrary, showed a distinct pattern towards higher radiosensitivity. Seventy-two percent of all mutation carriers were found to be radiosensitive (RIND score 1–3). Moreover, 28 % of *BRCA1* mutation carriers had RIND scores equal to 3 or 4, scores that were never observed in healthy volunteers. This simple scoring system can be valuable in assisting physicians in their decision-making in clinical follow-up and for refinement of radiotherapy at the individual level. Since our study is limited in sample size, a larger prospective study with blood samples of *BRCA1* mutation carriers will be undertaken to confirm and prove the importance of an in vitro radiosensitivity scoring system to assist clinical management of *BRCA1* mutation carriers.

Haploinsufficiency has been suggested as the main mechanism for hereditary breast carcinogenesis [45]. However, as NMD is not observed for mutations located in the 5′ and 3′ parts of the gene [46], a dominant-negative effect whereby the aberrant transcript abolishes the functionality of the WT allele cannot be excluded (for review, see [37, 47]). We wanted to evaluate if either or both mechanisms could influence the radiosensitivity score. Information on the stability of the mutant allele at the mRNA level was generated by both a fragment analysis and a novel massive parallel sequencing approach. To our knowledge, this is the first time that next-generation sequencing is applied to evaluate the level of NMD. The approach we describe here is straightforward and cost-effective for study of the relative expression of two alleles in a single individual.

The majority of patients included in this study are heterozygous for a mutation located in the central part of the gene leading to a PTC. These PTC-inducing mutations result in a truncated mRNA, which can be degraded by NMD. Generally, we observed that the mutated PTC allele was expressed at a ratio of 25–30 % of the WT allele at the mRNA level. Our results are consistent with the data of Anczuców et al. and Perrin-Vidoz et al. [46, 47]. Both articles describe a similar reduction of mRNA expression from PTC alleles in the central part of the gene and demonstrate the involvement of NMD in this decrease of mutant mRNA. A radiosensitive phenotype in 10 of 15 mutation carriers with a PTC allele undergoing NMD suggests haploinsufficiency as a mechanism leading to this phenotype in the large majority of the individuals.

Equal expression of the WT and mutant alleles at the mRNA level was observed in the patients with the start codon mutation (M01 and M02). For the patient with the c.212 + 3A > G mutation (M03), equal expression of a full-length transcript and a transcript lacking the last 22 nucleotides of exon 5 was observed in previous research by our group and confirmed by others [35, 36]. A higher median RIND score in the individuals carrying a mutation in the 5′ part of the gene, for which no NMD could be detected, compared with individuals with a PTC allele may point towards another mechanism involved in the radiosensitive phenotype, such as a dominant-negative effect. However, current knowledge on translation of mutant alleles not subjected to NMD into proteins is limited. Such detailed studies have not yet been undertaken; in most studies demonstrating a role for haploinsufficiency, *BRCA1* mutation carriers are compared as a group with non-carriers (e.g., [25, 38, 48]). Our data suggest the need for larger studies involving different types of mutations.

Unique to this radiosensitivity study compared with others are that we had access to material from several individuals from one family and that we could also evaluate the radiosensitive phenotype from several unrelated individuals carrying the same mutation. For four unrelated carriers of a Belgian founder mutation c.2359dup (p.Glu787fs*3), a large variation for the radiosensitive phenotype based on the RIND score was observed (range 0–4) (Table 2). Repeated assessments on blood specimens, taken on two different occasions from several individuals, ruled out that the different RIND scores were due to experimental variation. Given the smaller variation between related individuals, and because of the pleiotropic effect of IR on DNA (for example, strand breaks, fork stalling, base damage, DNA adducts [49–51]), we are convinced that other genetic factors influence the radiosensitivity. In previous research, for example, researchers have demonstrated the effect of SNPs on individual sensitivity to radiation therapy [52, 53]. In addition, our group has demonstrated the influence of RAD51, Ku70 and Ku80 SNPs as modulators of in vitro radiosensitivity in *BRCA1* mutation carriers and patients with BC [54, 55]. A larger study and the inclusion of a control population with relatives not harbouring the familial germline mutation could generate important insights.

## Conclusions

In our present study, using the G_2_ MN assay, we show that healthy individuals carrying a germline mutation in *BRCA1* are more radiosensitive than healthy control subjects after exposure to doses of 2 and 4 Gy. Seventy-two percent of the *BRCA1* mutation carriers showed a radiosensitive phenotype, and 28 % of *BRCA1* mutation carriers had high RIND scores of 3 or 4. Analysis of the mRNA stability of the mutant allele could not demonstrate a clear link between nonsense-mediated decay of the mutant allele and a radiosensitive phenotype. This, combined with the similar radiosensitive phenotype observed for related individuals but not for unrelated individuals carrying the same mutation, is indicative of the fact that additional genetic factors besides the *BRCA1* mutation may play a role in the radiation response. Our study emphasizes the need for large, prospective studies correlating the in vitro findings and exposure to radiation with the risk of developing breast cancer.

## Ethical approval and consent to participate

This study was approved by the ethics committee of the Ghent University Hospital (B670201111641 d.d. 20/09/2011). All study participants signed an informed consent form.

## Additional files

Additional file 1: Primers. The primer sequences (both forward and reverse). (PDF 29 kb) Additional file 2: Results of the fragment analysis. Illustration of fragment analysis data of an SNP without loss of the mutant allele. (PDF 176 kb) Additional file 3: Results of the fragment analysis. Illustration of fragment analysis data of an SNP with loss of the mutant allele. (PDF 227 kb)

## Abbreviations

A-T: ataxia-telangiectasia
BC: breast cancer
BN: binucleated cell
BrdU: 5-bromo-2′-deoxyuridine
CAF: caffeine
cDNA: complementary DNA
cDNAp: cDNA extracted in the presence of puromycin
CMGG: Centre for Medical Genetics, Ghent, Belgium
cRPMI: complete RPMI medium
cyto B: cytochalasin B
DSB: double-strand break
EDTA: ethylenediaminetetraacetic acid
FCS: foetal calf serum
gDNA: genomic DNA
HR: homologous recombination
IR: ionizing radiation
MN: micronucleus or micronuclei
NMD: nonsense mediated decay
PCR: polymerase chain reaction
PHA: phytohaemagglutinin
PTC: premature termination codon
RIND: radiosensitivity indicator
SD: standard deviation
SNP: single-nucleotide polymorphism
VAF: variant allele frequency
WT: wild type
