# Supplementary material for: Increased chromosomal radiosensitivity in asymptomatic carriers of a heterozygous BRCA1 mutation
Source: Breast Cancer Res. 2016 May 17;18:52. doi: 10.1186/s13058-016-0709-1 (PMC4869288; doi:10.1186/s13058-016-0709-1)
Supplement: Additional file 1: — Primers. The primer sequences (both forward and reverse). (PDF 29 kb) [file 13058_2016_709_MOESM1_ESM.pdf]

Additional file 1

Primers

Amplicon (exon)	Forward	Reverse
c.1A>G (E2)	TCTGCTCTGGGTAAAGGTAGTAGA	GAAGGCCCTTTCTTCTGGTT
c.1961del (E11)	GGAAGTCTTCTACCAGGC	TTAACTTCAGCTCTGGGAAAG
c.2311T>C (E11) = SNP	CCTAGCCTTCCAAGAGAAG	CCATGAATTAGTCCCTTGG
c.2359dup (E11)	CCTAGCCTTCCAAGAGAAG	CCATGAATTAGTCCCTTGG
c.3331_3334del (E11)	GTAGGTTCCAGTACTAATGAAG	CTGAAATCAGATATGGAGAGAAATC
c.3481_3491del (E11)	CCATATCTGATTTCAGATAACTTA	GATAAGTTCTCTTCTGAGGACTC
c.3661G>T (E11)	GCAGGAGTCCTAGCCCTTTC	GGTTACTGCAGTCATTTAAGCTATTC
c.4327C>T (E13)	GACTGCTCAGGGCTATCCTC	CAGACACCTCAAACTTGTCAGC
c.4391_4393delinsTT (E14)	GACTCTTCTGCCCTTGAGGA	AAGGGGATGACCTTTCCACT
