# Supplementary material for: Increased chromosomal radiosensitivity in asymptomatic carriers of a heterozygous BRCA1 mutation
Source: Breast Cancer Res. 2016 May 17;18:52. doi: 10.1186/s13058-016-0709-1 (PMC4869288; doi:10.1186/s13058-016-0709-1)

Additional file 2

Fragment analysis of c.2311T>C in BC01 (forward)
Limited loss of mutant mRNA could be detected (mean ratio = 0.7). This is in agreement with the MiSeq data with a VAF of approximately 50%.

a. cDNA

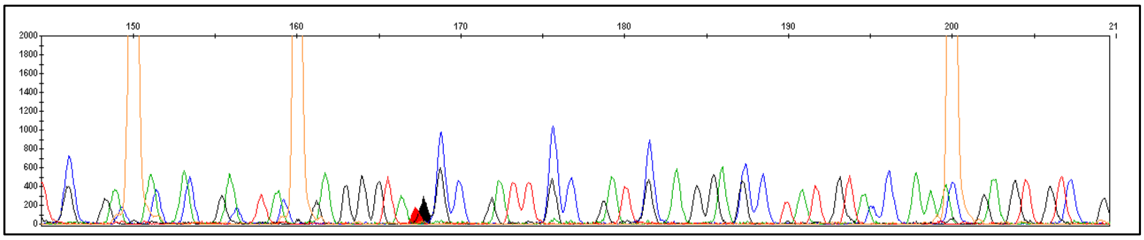

b. cDNA with puromycin

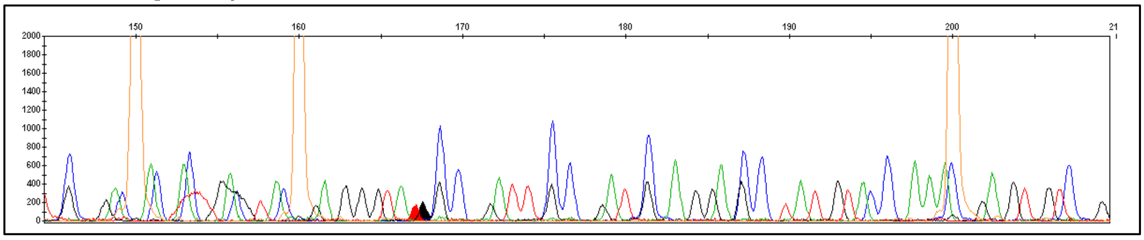

c. genomic DNA

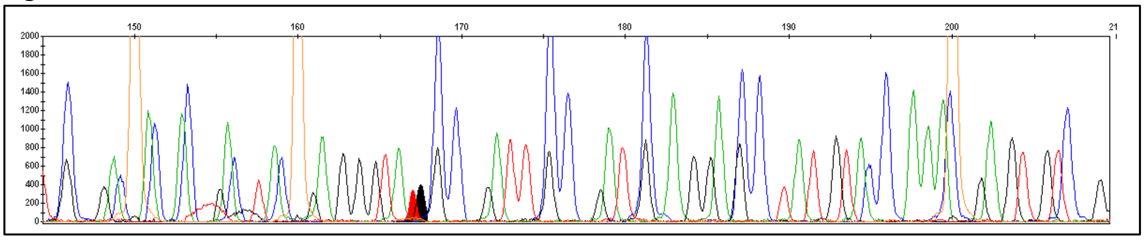

Supplement: Additional file 2: — Results of the fragment analysis. Illustration of fragment analysis data of an SNP without loss of the mutant allele. (PDF 176 kb) [file 13058_2016_709_MOESM2_ESM.pdf]
