# Supplementary material for: Increased chromosomal radiosensitivity in asymptomatic carriers of a heterozygous BRCA1 mutation
Source: Breast Cancer Res. 2016 May 17;18:52. doi: 10.1186/s13058-016-0709-1 (PMC4869288; doi:10.1186/s13058-016-0709-1)

Additional file 3:

Fragment analysis of c.2311T>C in BC17 (forward)
A loss of the mutant allele could be detected. In this patient, the T (black) represents the mutant allele. This loss of allelic imbalance is in agreement with the MiSeq data. The VAF for cDNA is 26%, or only half of the frequency on gDNA. This is reflected in the ratio of 0.48 for fragm. analysis in this particularly example.

a. cDNA

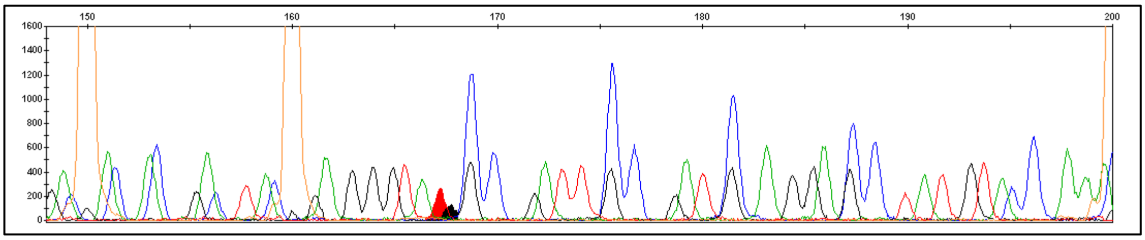

b. cDNA with puromycin

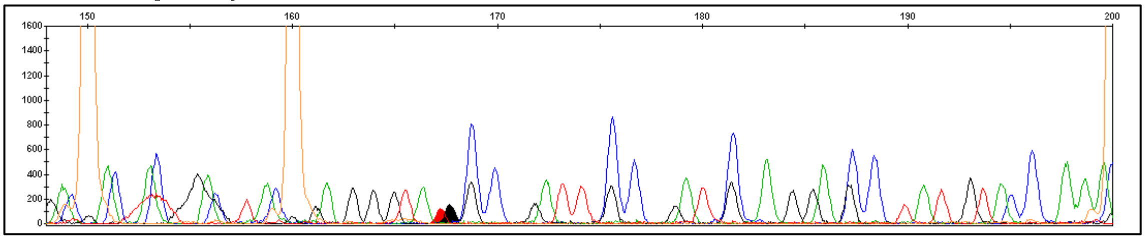

c. genomic DNA

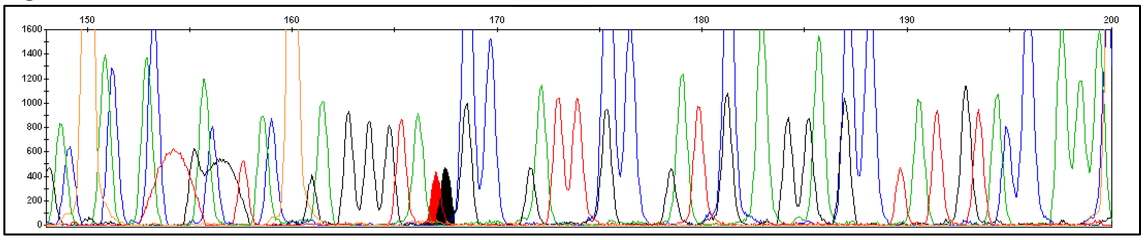

Supplement: Additional file 3: — Results of the fragment analysis. Illustration of fragment analysis data of an SNP with loss of the mutant allele. (PDF 227 kb) [file 13058_2016_709_MOESM3_ESM.pdf]
